# Interactive effects of biological, human and environmental factors on tick loads in Boran cattle in tropical drylands

**DOI:** 10.1186/s13071-021-04683-9

**Published:** 2021-04-06

**Authors:** Richard Chepkwony, Severine van Bommel, Frank van Langevelde

**Affiliations:** 1 Kenya Wildlife Service, P.O. Box 40241-00100, Nairobi, Kenya; 2 Strategic Communications Group, Wageningen University, P.O. Box 8130, 6700 EW Wageningen, The Netherlands; 3 Wildlife Ecology and Conservation Group, Wageningen University, P.O. Box 47, 6700 AA Wageningen, The Netherlands; 4 School of Agriculture and Food Sciences, University of Queensland, Gatton, QLD 4343 Australia; 5 School of Life Sciences, Westville Campus, University of KwaZulu-Natal, Durban, 4000 South Africa

**Keywords:** Tick-borne diseases, Wildlife, Boran cattle, Commercial ranches, Transhumance, Tropical areas

## Abstract

**Background:**

Tick-borne diseases (TBDs) are a serious threat to humans, wildlife and livestock, and cause severe economic losses in many tropical drylands. The effective control of TBDs has been constrained by limited understanding of what determines tick loads in animals. We tested interactive effects of several biological factors (sex, age and body condition), one environmental factor (rainfall) and one human factor (management type) on tick loads in animals.

**Methods:**

We collected ticks on animals at four sampling sites in the semi-arid savanna area of Laikipia County, Kenya, of which two are commercial ranches and the other two are open pastoral grazing areas. From 2017 to 2019, we collected a total of 2038 ticks from 619 domestic animals from various cattle and camel herds and from 79 tranquilised wild animals.

**Results:**

Generally, wild herbivores (zebras, rhinos and elephants) had higher tick loads than domestic animals. As 83% of the tick samples were taken from Boran cattle, we analysed tick load in these cattle in more detail. Boran cattle had high tick loads in the wet season, especially those animals in poor condition. No differences were found between female and male cattle, regardless of season. The calves had high tick loads during both the wet and dry seasons, whereas the sub-adult and adult cattle had less ticks during the dry season. Cattle on the intensively managed ranches had lower tick load than those in the transhumant management system.

**Conclusion:**

These findings highlight the importance of establishing effective control of ticks on domestic animals in transhumant management systems as tick loads were high on these animals in both the wet and dry season.

**Graphic abstract:**

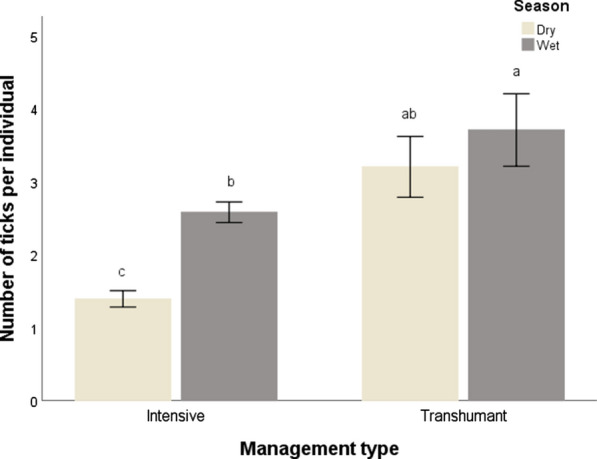

## Background

Tick infestation in animals is one of the major challenges in tropical drylands related to animal and human health and causes diseases with substantial economic losses [1, 2]. Ticks transmit pathogens that can cause diseases such as East Coast fever, anaplasmosis, babesiosis and tick-borne encephalitis, leading to mortalities in livestock, wildlife and humans [3, 4]. It is estimated that tick-borne diseases (TBDs) cause approximaely 10–80% of livestock mortalities in Africa, affecting many livelihoods [1, 5]. Control of TBDs by farmers in tropical drylands is difficult due to the interactions of human, biological and environmental factors [6]. These factors can influence tick load in host animals due their effects on the reproduction of ticks [6], the host-seeking strategies of the ticks [7] and the efficacies of tick control [2, 8]. Furthermore, tick infestation level in animals is the focal point for the control of TBDs [5, 9]. Hence, it is crucial to estimate which animal species have high tick infestation levels and identify factors influencing these differences between species in order to plan for effective control [10, 11]. Many studies on the risk of TBDs have mainly focussed on several factors, such as environmental (rainfall, temperature and humidity) [12], biological (age, sex, body condition) [13, 14] and human factors (land use, animal husbandry) [15–18]. However, the interactive effects of biological, environmental and human factors on tick loads in animals are poorly understood [6, 19, 20].

It has been established that changes in rainfall influencing humidity and temperature may influence tick load in animals [21]. For example, moderate rainfall and high humidity provide conductive micro-climatic conditions for mass reproduction of ticks and subsequent infestation in animals [10]. We therefore expect a higher tick load in animals in the wet season than the dry season (hypothesis 1). Studies have shown that biological factors, such as age, sex and body condition of the host animals, influence tick load [22, 23]. As the two seasons are important drivers of tropical drylands, we tested the interactive effects of each of the biological factors and management type with season. For example, adult animals may face a lower risk of infection by pathogens due to lower tick loads because of their good body condition [11]. We therefore expect animals in a poor condition to have a higher tick load than those with a good body condition, especially during the wet season (hypothesis 2). Moreover, lactating female hosts may have a poor body condition due to higher net energy spent on breeding and are more prone to tick infestation than males or non-lactating females. We thus expect females, in general, to have more ticks than males, especially during the wet season (hypothesis 3). Several hypotheses have predicted that calves will carry higher tick loads than adult hosts [24, 25], either because (i) adult hosts develop immunity and/or behavioural adaptations to avoid or remove parasites, or (ii) heavily infested calves die before adulthood (i.e. the selection hypothesis). We thus expect calves to have more ticks than sub-adults and adults, especially during the wet season (hypothesis 4).

In many tropical drylands, fences have been widely used to delineate property ownership boundaries and other human activities such as farming, influencing the movement of host species [26, 27]. For example, studies have shown that the large-scale movement of animals during dry seasons (transhumance) in the search for scarce water and pasture increases the chances of either spreading or even introducing new tick species in areas, thereby increasing the risks of the spread of diseases [28, 29]. Furthermore, in intensively managed production systems, farmers frequently apply chemicals to control tick load in animals. For example, studies in Kenya and Tanzania showed that frequent chemical control of ticks in domestic animals also benefited wild animals [30, 31] and reduced tick densities in vegetation [5, 18, 28, 32]. Conversely, failure to control ticks has been shown to increase tick load in hosts, aggravating the risks of pathogen spread [7, 33, 34]. We therefore expect lower tick loads in intensively managed systems compared to transhumant management systems, especially during the wet season (hypothesis 5). Given the wide range of determinants potentially affecting tick burden in animal hosts, a clear understanding of the interaction effects of these factors is paramount for their effective control and remains an important knowledge gap in the emerging field of infectious disease ecology [20, 35, 36].

## Methods

### Study area

**Fig. 1:**
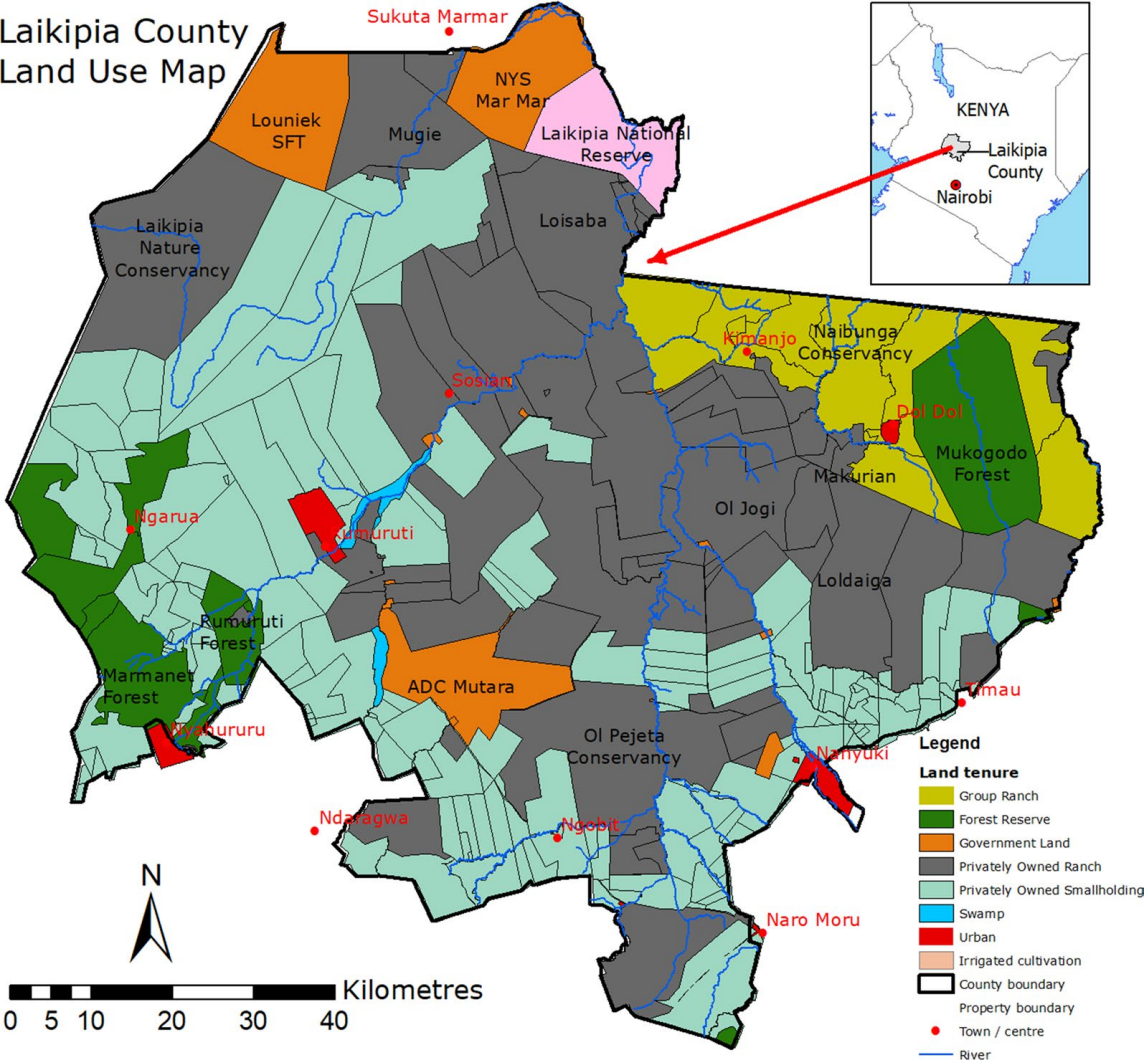
Land use map of Laikipia. The study sites were Olpejetas and surrounding areas, Loldaiga and Naibunga-Makurian (light green) and Segera-Endana (light blue) to the north of Olpejeta Conservancy

This study was conducted at sites in Laikipia County located between 0°04'60.00"N and 36°39'59.99"E in Kenya [26] (Fig. 1). The county has an area of approximately 10,000 km^2^ with high densities of wild and domestic animals [17]. The average annual rainfall in the county varies between 400 and 750 mm, with higher precipitations in the areas bordering the Aberdare ranges and Mt. Kenya [30]. The short rains occur in October and November, while the long rains occur from March to May [26]. The temperature ranges between 16 °C and 26 °C, with the low-lying areas in the north being generally hotter [26]. The farms are managed as either open or fenced, and their sizes vary and range from a few acres to ≥ 100, 000 acres [27, 37].

The county regularly experiences high immigration rates of transhumance livestock from pastoral communities, such as the Pokot, Samburu, Borana, Turkana and the non-resident Maasai, due to prolonged droughts. Some of the common wildlife species in the area include the African elephant (*Loxodonta Africana*), African buffalo (*Syncerus caffer*), black rhino (*Diceros bicornis*), white rhino (*Ceratotherium simum*), eland (*Taurotragus oryx*), impala (*Aepyceros melampus*), common zebra (*Equus burchellii*), Grevy’s zebra (*Equus grevyi*), bushbuck (*Tragelaphus scriptus*), waterbuck (*Kobus defassa*) and lion (*Panthera leo*) [30, 38]. Common domestic animals include cattle breeds, such as Boran (*Bos indicus*), Aberdeen Angus (*Bos taurus)* and Ankole (*Bos taurus africanus*). Detailed descriptions of common wild and domestic animals in the study area are provided by Young et al. [38].

### Sampling sites

We used four sampling sites to collect ticks on animals in Laikipia County, of which two are commercial ranches and the other two are open pastoral grazing areas. The two distinct livestock management systems in the area differ in the intensity of acaricide applications and animal husbandry, with possible implications on tick infestation levels in animals [39]. The commercial ranches are the Olpejeta Conservancy and Loldaiga Hills Conservancy, while the community areas are Naibunga-Makurian and Segera-Endana (Fig. 1). The two commercial properties have well-maintained fences and integrate wildlife conservation and livestock ranching as their core activities. The two community grazing areas are open and allow unrestricted movement of wildlife and domestic animals [38, 40]. These study sites are located in two important movement corridors linking Mt. Kenya to the east and the Aberdare ranges to the west. The two mountain ranges have formed traditional dry season grazing refuges for wild and domestic animals.

### Data collection

#### Host species

The study was conducted from February 2017 to September 2019 with the aim to determine the tick loads in domestic animals and wildlife. We investigated cattle of different management systems, constituting exotic, cross and local breeds: Boran, Aberdeen Angus and Ankole of different sex, age and body condition. We also investigated camels (*Camelopardalis dromedarii*), which were sampled in spraying races or temporary holding pens in pastoral areas. For wild animals, we collected ticks in the black rhino, elephant, lion and Grevy’s and Burchell’s zebra during wildlife translocation or treatment of sick animals by the Kenya Wildlife Service (KWS) veterinary teams. We collected ticks in lions because they have a wide predator–prey relationship with herbivores and may have a variety of ticks from different hosts [41].

Generally, farmers in the area categorise the age of cattle as either: calf (0–9 months), sub-adult (9–36 months) or adult (> 36 months). The wild animal hosts were also categorised as either calf, sub-adult or adult. The age categories for the different wildlife species were estimated based on: (i) the relative wither height for zebra (*E. burchellii*) [42] and the African elephant (*Loxodonta africana*; https://www.elephantvoices.org); (ii) whiskers, dental structure and mane appearance for the lion (*Panthera leo*; http://agingtheafricanlion.org); and (iii) the black rhino monitoring records from Olpejeta Conservancy to estimate the age categories for rhino. We scored the body condition of both domestic and wild hosts as either poor, fair or good, based on Heinrichs and Ishler [43]. We treated the management systems as either intensive (fenced areas) or transhumance (open grazing pastoral). The mode of chemical applications was classified as: (i) high-pressure spray race nozzles (for intensive management system); (ii) portable hand-sprays (for transhumance systems); or (iii) none (for wildlife).

#### Tick sampling

Prior to collecting ticks on cattle, we obtained consent from the farmers and research authorisation from KWS. Three collectors and one enumerator were deployed to count and record tick sampling details. The tick collectors stood on either side of a spray race or temporary cattle holding pen to optimise tick checking and collection. The ears, brisket (dewlap in the case of cattle), groin region, tail, belly and neck region were examined for the presence of ticks [44]. All visible ticks were collected through either hand-picking or the use of forceps. Each tick specimen were then placed in a vial with 70% ethanol and labelled with a unique sample ID that comprised the farm ID or locality, host species/breed and body location. A similar approach was used to collect ticks in tranquilised wildlife.

Since ticks are notoriously difficult to accurately identify in the field, we resorted to temporarily identifying them based on their morphology, colour or names used by the local pastoral farmers or workers who are adept at tick species description. The specimens were transported to the KWS forensic laboratory (Nairobi, Kenya) for morphological identification under a dissecting stereomicroscope (Leica DM500 microscope, Hach, USA). Ticks were identified following the available taxonomic keys and the monographs of the ticks of Kenya in accordance with Walker et al. [44]. We updated our data with the correct tick species names.

#### Rainfall data

Rainfall data were obtained from the rain gauges located at the Olpejeta Conservancy and Loldaiga Hills Conservancy. The rainfall data were collected on a daily basis at the two commercial ranches for routine range management. Since the community areas had no rain gauges, we used rainfall data from the two adjacent conservancies to represent rainfall amounts in the respective community areas. We used the minimum rainfall in the two areas and set the lower limit of < 50 mm of rainfall as ‘dry’ for 2 or 3 consecutive months and > 100 mm as ‘wet’ over a similar duration.

### Data analysis

The total number of ticks per host species was used as the dependent variable. The explanatory variables were season, sex, age, body condition of the host and management type for tick control. We first performed an exploratory data analysis following the protocol described in Burnham and Anderson [45]. We used the Poisson generalised linear model (G_Z_LM) with a log-link function, which is appropriate for count data. We tested all of the main and two-way interactions of the explanatory variables. Differences between groups were tested using multiple pairwise comparisons (Sidak test) [45]. All analyses were performed in IBM SPSS Statistics version 25 (IBM Corp., Armonk, NY, USA).

## Results

A total of 698 domestic and wild animals (577 Boran cattle, 2 Ankole cattle, 11 Aberdeen Angus cattle, 6 Cherokee cattle, 23 camels, 19 elephant, 28 black rhino, 3 lion, 17 Grevy’s zebra, 12 Burchell’s zebra) were examined for ticks. Of all the animals sampled for ticks, 53.2% were female and 46.8% were male, comprising 62.9% adults, 24.1% sub-adults and 13% calves.

**Fig. 2:**
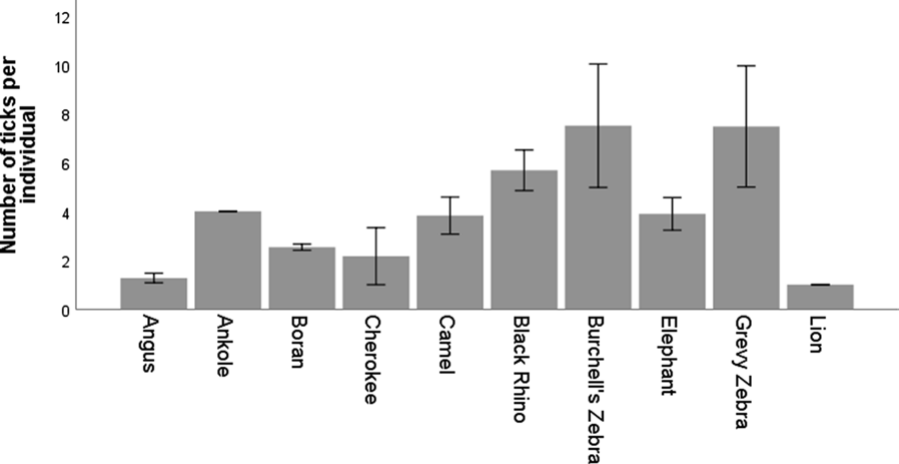
Mean tick load per individual for the different host species. The error bars represent the standard error. Angus, Ankole, Boran and Cherokee are breeds of cattle. Host species (*X*-axis) are presented from left to right as first domestic animals and then the wild species. See text for the total number of animals per host species. The minimum–maximum number of ticks per host species are: Angus (3–20), Ankole (2–2), Boran (0–39), Cherokee (1–2), camel (1–13), black rhino (1–14), Burchell's zebra (1–6), elephant (0–3), Grevy’s zebra (0–43) and lion (1–6)

In total, 2038 adult Ixodid ticks (female: 1053; male: 985) were collected. They belonged to three genera:* Rhipicephallus* (88.4%),* Amblyomma* (6%) and* Hyalomma* (5.6%). The ticks in these three genera comprised 17 positively identified tick species, of which the most common were: *Rhipicephallus evertsi*,* R. pulchellus*,* R. decoloratus*,* R. appendiculatus*,* Hyalomma dromedarii* and *H. rhinocerotis*. We also collected rare species:* R. camicasi*,* R. pravus*,* H. scupense*,* R. rufipes*, *H. lusitanicum* and *R. sanguines*.* Rhipicephallus evertsi*,* R. pulchellus*, *R. decoloratus*, *R. appendiculatus* and *H. trancatum*; these were found in almost all of the hosts sampled (generalists). *Amblyomma rhinocerotis* and *A. coherence* were specific to black rhino, while *R. camicasi* was specific to camel. However, *H. dromerdarii*, known to be specifically hosted by camel, was also found in Boran cattle. The mean number of ticks per animal was 2.9 ± 0.1, regardless of the season (see Fig. 2 for the division over the animal species; see the data for the tick species per animal host). As some host species had very low sample sizes, we compared the tick load in all domestic species with that in all wild herbivore species (zebras, rhinos and elephants). The wild herbivore species had higher tick load per individual than the domestic species (Mann–Whitney U-test: *U* = 18,825.5, *N*_wild_ = 102, *N*_domestic_ = 596, *P* < 0.001).

**Fig. 3:**
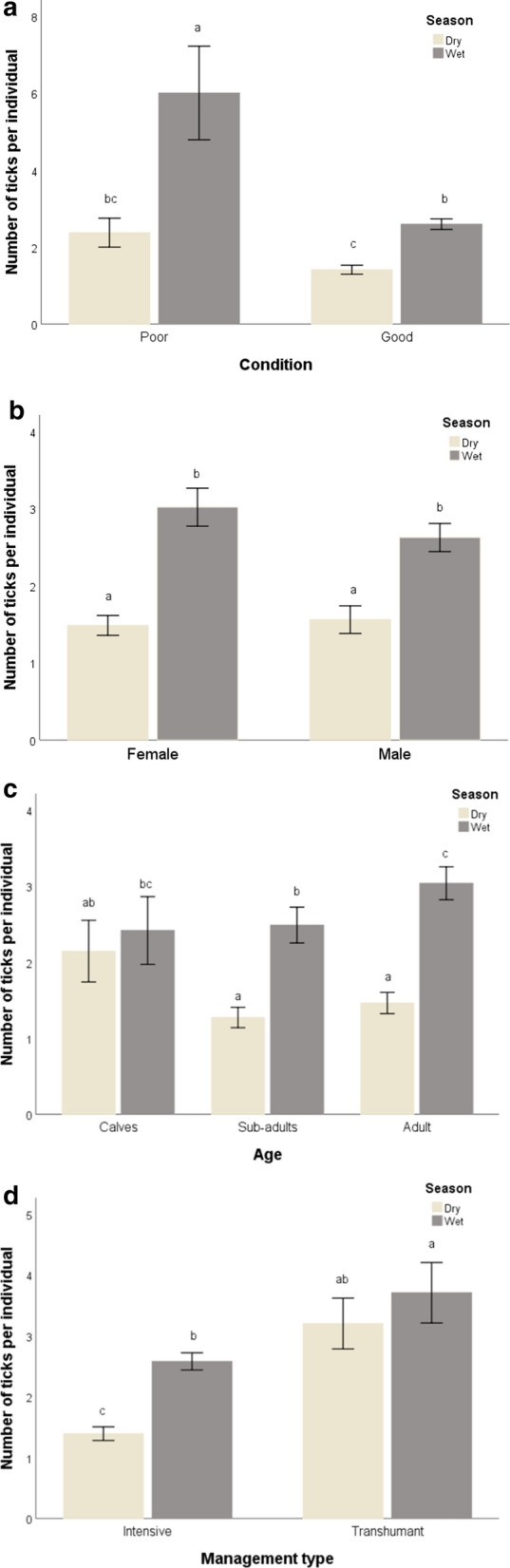
Differences in tick load in Boran cattle between body condition and season (**a**), sex and season (**b**), age and season (**c**), management type and season (**d**). Different locase letters indicate significant differences. Bars show mean ± standard error

For the remaining analyses, we only considered ticks on Boran cattle as the other cattle species had low sample sizes: 83% of the samples were taken from Boran cattle. Overall, tick load in Boran cattle was significantly higher during the wet season than during the dry season. We found a difference in tick load between animals in poor and good body condition, but there was no interactive effect of body condition and season (Table 1; Fig. 3a). Animals in poor body condition had the highest tick loads. Also, we did not find a difference between females and males or an interactive effect of sex and season on tick load in Boran cattle (Table 1; Fig. 3b). We found significant differences in tick load between calves, sub-adults and adult hosts and a significant interaction with season (Table 1; Fig. 3c). During the dry season, the adults and sub-adults had lower tick loads than calves. The interaction between season and management type was also significant (Table 1; Fig. 3d). The tick load in the cattle on the intensively managed ranches was lower during both the dry and wet seasons. Cattle in the transhumant management systems had the highest tick load during the wet season, but the difference with the dry season was not significant.

**Table 1:** Results of the generalised linear model for tick load (number of ticks/individual) on Boran cattle as response variables

Model	Explanatory variables	Wald Chi-square	AIC	*df*	*P*
1	Season	61.96		1	< 0.001
	Body condition	48.40		1	< 0.001
	Season × body condition	2.67	2527.4	1	0.102
2	Sex	0.35		1	0.550
	Season	66.02		1	< 0.001
	Sex × season	1.56	2649.5	1	0.211
3	Age	6.18		2	0.046
	Season	39.23		1	< 0.001
	Age × season	9.97	2639.4	2	0.007
4	Season	14.29		1	< 0.001
	Management system	35.05		1	< 0.001
	Season × management system	5.41	2606.6	1	0.020

Each model is numbered. The table specifies for each explanatory variable in the model (including the interaction between two explanatory variables) the test statistic Wald Chi-square and the corresponding *P* values, and for the model the Akaike Information Criterion (AIC). For all models, *N* = 571

## Discussion

The aim of our study was to identify tick species in wild and domestic hosts and the determinants of the observed tick loads. Ultimately, our analysis of the biological, environmental and human factors determining tick load could only be done for the domestic Boran cattle. We found interactive effects of season, host characteristics (age, sex and body condition) and the management system on tick loads. Tick load in Boran cattle in Laikipia was generally low compared to findings from other areas, such as Tanzania, Ethiopia [46] and Uganda [47], possibly due to the massive application of acaricides by some farmers in Laikipia. For example, a high prevalence of up to 82% of ixodid ticks was reported in domestic transhumance cattle from different parts of Ethiopia [46]. The high tick infestations in many parts of Ethiopia are also attributed to poor tick control strategies and the large-scale transhumance movement of livestock in search of water and pasture during the dry season [46]. The relatively low tick loads in our study seem to align with tick loads reported in other studies in the area that found low tick count in the vegetation [18]. We acknowledge that visual inspection may be limited; to prevent this as much as possible, we conducted pre-counting inspection and validation to ensure consistency in counting and observer bias. Moreover, we used domestic animal herders who are adept at tick observation. Although we found relatively low tick loads on the host species, mortalities due to tick-borne diseases are considerable [21].

The results of this study suggest that there were more ticks on Boran cattle during the wet season (support for hypothesis 1); in particular, animals in poor condition in the wet season had a high tick load (support for hypothesis 2). The wet season conditions in tropical drylands are characterised by moderate to high rainfall intensity, increased humidity, increased vegetation cover and increased presence of suitable hosts [5, 48]. Compared to the dry season, the wet season provides more conducive micro-climatic conditions for the mass reproduction and distribution of ticks in hosts [6]. Chepkwony et al. [21] report that cattle mortalities due to tick-borne diseases (East Coast fever or anaplasmosis) are higher during the wet season after a few months of drought. The months of drought lower the body condition of the animals due to limited forage and water, exacerbating their tick loads [28, 49], resulting in mortalities. In contrast, VanderWaal et al. [48] found that in Kenya the parasite load, such as ticks, fleas and mites, was more often shared at watering points during the dry season than during the wet season [12, 48]. Seasonality and body condition are important determinants for tick loads in animals due to the biology and behaviour of the ticks and their hosts, impacting on pathogen transmission [23, 28, 48, 49].

Sex-biased differences in parasite intensity are commonly observed [50], with studies suggesting that males are often more likely to come into contact with ticks than females due to behavioural or physiological differences, as exemplified in chipmunks [14, 30] and domestic cattle in Ethiopia [46] and rodents [14]. These findings are in contrast to our observations of no differences in parasite intensity between females and males, regardless of season (no support for hypothesis 3). In contrast to the absence of differences between males and females in Boran cattle, calves had high tick loads during both the wet and dry season, whereas the sub-adult and adult cattle had less ticks during the dry season (support for hypothesis 4). Several hypotheses [24, 25] predict that calves will carry heavier tick loads than adult hosts, either because (i) adult hosts develop immunity and/or behavioural adaptations to avoid or remove parasites and/or (ii) heavily infested calves die before adulthood (i.e. the selection hypothesis).

Cattle on the intensively managed ranches had lower tick loads than the transhumance management system (support for hypothesis 5). The intensive management systems use acaricides [39] and generally have fences to limit host movements; both measures reduce tick loads on cattle. Conversely, transhumance, which is an important adaptation for pastoralist communities, has been shown to positively influence parasite spread and disease dynamics [31], as livestock from surrounding areas may probably import ticks [18, 40]. The results of this study thus provide empirical evidence that tick loads in animals at intensive management system had lower tick loads than those in transhumant management systems.

To our knowledge, this is one of the few studies in tropical drylands that has quantified the role of season, biological factors, management type and their interactions in determining tick loads in animal species. To develop models that can be used to predict, design or implement tick control measures and mitigate future TBDs infections [51, 52], there is a profound need to better understand the interaction effects of these biological, human and environmental factors on tick load in hosts [18, 20, 35]. The findings of this study increase our understanding of tick–host–pathogen interactions, a fundamental prerequisite for effective control of ticks. The findings highlight the importance of establishing effective control of ticks in domestic animals in transhumant management systems as tick loads were high in these animals during both the wet and dry seasons. For effective control of ticks in tropical drylands, we need an integrated approach that includes the involvement and co-ordination of farmers, veterinary officials, wildlife managers, environmentalists, acaricide manufacturing companies and chemical regulatory authorities. The integrated approach may increase the space for information and knowledge sharing, which may enhance the decision-making process by famers and other actors for effective tick control under the prevailing human and environmental conditions in an area.

## Data Availability

After acceptance, the data will be made available through a repository.
